# STING activator 2′3′‐cGAMP enhanced HSV‐1‐based oncolytic viral therapy

**DOI:** 10.1002/1878-0261.13603

**Published:** 2024-02-23

**Authors:** Patricia Angela Sibal, Shigeru Matsumura, Toru Ichinose, Itzel Bustos‐Villalobos, Daishi Morimoto, Ibrahim R. Eissa, Mohamed Abdelmoneim, Mona Alhussein Mostafa Aboalela, Nobuaki Mukoyama, Maki Tanaka, Yoshinori Naoe, Hideki Kasuya

**Affiliations:** ^1^ Cancer Immune Therapy Research Center, Graduate School of Medicine Nagoya University Japan; ^2^ Department of Surgery II, Graduate School of Medicine Nagoya University Japan; ^3^ Faculty of Science Tanta University Egypt; ^4^ Department of Microbiology, Faculty of Veterinary Medicine Zagazig University Egypt; ^5^ Medical Microbiology and Immunology Department, Faculty of Medicine Zagazig University Egypt; ^6^ Department of Otolaryngology Graduate School of Medicine Nagoya University Japan; ^7^ Takara Bio Inc. Kusatsu Japan

**Keywords:** 2′3′‐cGAMP, HSV1, oncolytic virus, pancreatic cancer, STING

## Abstract

Oncolytic viruses (OVs) can selectively replicate in tumor cells and remodel the microenvironment of immunologically cold tumors, making them a promising strategy to evoke antitumor immunity. Similarly, agonists of the stimulator of interferon genes (STING)‐interferon (IFN) pathway, the main cellular antiviral system, provide antitumor benefits by inducing the activation of dendritic cells (DC). Considering how the activation of the STING‐IFN pathway could potentially inhibit OV replication, the use of STING agonists alongside OV therapy remains largely unexplored. Here, we explored the antitumor efficacy of combining an HSV‐1‐based OV, C‐REV, with a membrane‐impermeable STING agonist, 2′3′‐GAMP. Our results demonstrated that tumor cells harbor a largely defective STING‐IFN pathway, thereby preventing significant antiviral IFN induction regardless of the permeability of the STING agonist. *In vivo*, the combination therapy induced more proliferative KLRG1‐high PD1‐low CD8^+^ T‐cells and activated CD103^+^ DC in the tumor site and increased tumor‐specific CD44^+^ CD8^+^ T‐cells in the lymph node. Overall, the combination therapy of C‐REV with 2′3′‐cGAMP elicited antitumor immune memory responses and significantly enhanced systemic antitumor immunity in both treated and non‐treated distal tumors.

## Graphical abstract

The cell‐impermeable 2′3′‐cGAMP activates the cellular antiviral STING pathway. Here, we found that due to the defective STING pathway of tumor cells and their lack of 2′3′‐cGAMP receptor, 2′3′‐cGAMP treatment mainly affected immune cells and had negligible effect on the tumoral cytotoxicity of the oncolytic virus C‐REV. Combining C‐REV and 2′3′‐cGAMP drastically decreased tumor burden and promoted immune memory formation.

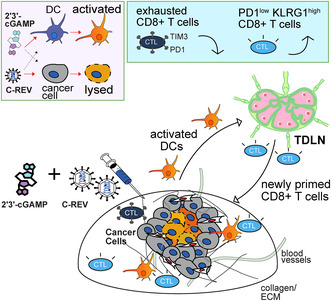

**Abbreviations**
APC: antigen‐presenting cells
cGAMP: cyclic guanosine monophosphate–adenosine monophosphate
CR: complete response
C‐REV: Canerpaturev
DC: dendritic cells
dsDNA: double stranded DNA
E‐FTV: expected fractional tumor volume
ENPP1: ecto‐nucleotide pyrophosphatase phosphodiesterase 1
FTV: fractional tumor volume
GM‐CSF: granulocyte‐macrophage colony‐stimulating factor
hpi: hours post‐infection
HSV: herpes simplex virus
ICI: immune checkpoint inhibitor
IFN: interferon
IL: interleukin
IRF3: interferon regulatory factor 3
IT: intratumoral
LDH: lactate dehydrogenase
MHC: major histocompatibility complex
MOI: multiplicity of infection
MTT: 2,5‐diphenyl‐2H‐tetrazolium bromide
NK: natural killer cells
O‐FTV: observed fractional tumor volume
OV: oncolytic virus
PD‐1: programmed cell death protein‐1
PDAC: pancreatic ductal adenocarcinoma
PDL‐1: programmed death ligand‐1
SCC: squamous cell carcinoma
STING: stimulator of interferon genes
TBK 1: TANK binding kinase 1
TCR: T‐cell receptor
TDLN: tumor drained lymph node
TIL: tumor‐infiltrating lymphocytes
TME: tumor microenvironment
T‐VEC: Talimogene Laherparepvec

## 1 Introduction

As of this date, pancreatic ductal adenocarcinoma (PDAC) remains one of the most aggressive solid malignancies, with a 5‐year survival rate of only 8% worldwide [1]. PDAC is an epithelial neoplasm that develops in the exocrine compartment, which accounts for 90% of the pancreas and is responsible for synthesizing and secreting digestive enzymes. Its high resistance against the three primary cancer treatment modalities – surgery, chemotherapy and radiation therapy – has been well established. These limitations has driven the development of immunotherapies, which seek to enhance an individual's antitumor immune response by promoting tumor recognition or improving the effector function of immune cells [2].

A front runner in this field is immune checkpoint blockade (ICB) therapy, which utilizes antibodies that block immune checkpoint molecules to limit effector immune cell exhaustion. Although ICB significantly increased prognostic outcomes in several types of solid cancers, it provided only marginal benefits for PDAC patients [3, 4, 5]. Such resistance has been attributed to PDAC's unique, highly immunosuppressive tumor microenvironment (TME). Compared with other solid tumors, PDAC forms stiff solid tumor tissues by substantial crosstalk between the tumor cells and stromal fibroblasts [6, 7]. This high fibrotic stiffness then functions as a barrier that limits the infiltration of effector T‐cells [8]. Although ICB can minimize exhaustion by countering the abundant immune checkpoint signaling molecules such as PDL‐1 expressed by the tumors and surrounding suppressive macrophages, dendritic cells and regulatory T‐cells in the PDAC TME, ICB is rendered ineffective if there is only a minimum number of CD8^+^ T‐cells to act on [5, 9]. Indeed, numerous clinical trials have demonstrated that the clinical effectivity of ICB was correlated with the density of pre‐existing CD8^+^ T‐cells in the tumor site [5, 9]. Considering that oncolytic viruses (OVs), another type of immunotherapy, have been reported to increase infiltration of effector CD8^+^ T‐cells, the combination therapy of OVs with ICB against PDAC is being explored in clinical trials [10].

Oncolytic viral (OV) therapy involves the use of attenuated viruses that selectively replicate in tumor cells. OV infection solely depends on the presence of the viral entry receptors on the surface of tumor cells. As such, lysis of tumor cells occurs effectively regardless of the surface expression of major histocompatibility complex (MHC) Class I, making OV therapy ideal for tumor types with minimal expression of MHC Class I, as in PDAC [11, 12, 13]. Alongside this, OV treatment triggers the release of inflammatory cytokines, which then evokes an acute inflammation capable of reprogramming the TME from immunosuppressive to immunogenic. In doing so, OVs enhance the recognition of tumor‐specific antigens by the immune system [14].

Several kinds of OVs are currently under investigation in clinical studies on various cancers, including pancreatic cancer [14, 15, 16]. A Herpes Simplex Virus Type‐1 (HSV‐1)‐based OV, talimogene laherparepvec (T‐VEC, Imlygic™, Amgen, Cambridge, UK), armed with granulocyte‐macrophage colony‐stimulating factor (GM‐CSF) was the first OV to be approved by the US, EU, and Australia for clinical use [17]. Aside from T‐VEC, another promising HSV‐1‐based OV is Canerpaturev (C‐REV, formerly known as HF10), which is a spontaneously occurring HSV‐1 mutant that lacks functional expression of UL43, UL49.5, UL55 and UL56 [18]. Our previous study using the squamous cell carcinoma model revealed that C‐REV treatment was able efficiently to recruit the CD8 T‐cells with a low expression of PD‐1 to the tumor tissues, suggesting that C‐REV has the potential to overcome the limitations posed by the PDL‐1 enriched immunosuppressive TME characteristic of aggressive solid malignancies [19]. In addition, we also confirmed the efficacy and safety of C‐REV with unresectable PDAC in phase I dose‐escalation clinical trials [20, 21].

Like most OVs, the clinical safety of the C‐REV is highly dependent on cellular viral detection mechanisms, which ensures C‐REV tumor‐selective replication. The STING pathway has long been regarded as the principal cellular antiviral host defense system. It has several sensor molecules for detecting pathogens, including cGAS, which functions as a sensor for short double‐strand DNA (dsDNA), typical of many viral DNAs [22, 23]. Upon binding with dsDNA, cGAS catalyzes the synthesis of cyclic GMP‐AMP (2′3′‐cGAMP) from ATP and GTP. In turn, 2′3′‐cGAMP binds to STING and induces its activation and subsequent translocation from the ER to the ER‐Golgi intermediate compartment and the Golgi apparatus [22, 23, 24]. STING then forms a clustered structure with the recruited TBK1, which then becomes self‐activated via autophosphorylation. Once activated, TBK1 phosphorylates IRF3, which then forms dimers upon phosphorylation and subsequently translocates into the nucleus to start the rapid transcription of the type‐I interferons (IFN) crucial for antiviral responses [22, 23].

Once the STING pathway of non‐malignant cells detects the viral DNA of OVs, OV replication is inhibited. As for tumors, the integrity of the STING pathway varies depending on the stage of tumor formation. In the initial stages, tumor cells still harbor an intact STING pathway, allowing cGAS to detect self‐DNA fragments leaking from the nucleus as the genome undergoes compelling rearrangement [25, 26]. The released IFN then induce apoptosis and attract immune cells capable of attacking the tumor cells, preventing tumor progression. Conversely, advanced tumor cells carry numerous defects in the STING pathway. Although these defects have been critical in the tumor cell's evasion of the immune system, such defects make advanced tumors ideal environments for OV replications.

Considering how the increased activation of the STING pathway was expected also to increase antiviral responses, combination therapy of STING agonists and OVs remains largely unexplored despite the immunostimulatory potential of STING agonists. High doses of STING agonists were found to induce apoptosis in tumor cells, whereas low to moderate doses of STING agonists activated dendritic cells, leading to increased cross‐priming efficiency [27]. Our previous work has also shown that the STING pathways of advanced pancreatic tumor cell lines are defective enough such that negligible type‐I IFN are induced even when treated with 2′3′‐cGAMP, a membrane‐impermeable STING agonist [28]. In this study, we administered 2′3′‐cGAMP on the tumor site and hypothesized that it would mainly activate the STING pathway in non‐malignant cells, particularly immune cells in the tumor tissue, instead of activating the STING pathway inside the tumor cells. Our results revealed that 2′3′‐cGAMP treatment did not inhibit C‐REV cytotoxicity against tumor cell lines *in vitro*. Combination therapy conferred synergistic antitumor efficacy by inducing a significant population of cross‐presenting DC and terminally differentiated CD8^+^ T‐cells with low PD1 expression *in vivo*.

## 2 Materials and methods

### 2.1 Tumor challenge and treatments

For animal experiments, all mice were maintained under specific pathogen‐free conditions. Mice were randomly divided into treatment groups before therapy, and all mice were treated and evaluated in the same way; experimenters were not blinded. Samples were excluded from the final analysis only when a mouse developed health problems unrelated to the treatment, following animal care guidelines. All experiments were reviewed and approved by the Animal Care University Committee following the Guidelines for Animal Experimentation at Nagoya University (M210766‐001). All mice were observed daily and were euthanized when they showed signs of morbidity. Four to eight animals per group were used for antitumor efficacy studies.

Female C3H/HeN (C3H) or C57BL/6J mice 6–7 weeks old were purchased from Japan SLC (Hamamatsu, Japan). Tumors were cut into 2 mm^3^ cubes. One tumor cube was inoculated into each flank (right and left). When the average tumor size reached 100 mm^3^ in each flank, mice were randomly divided into four groups, namely, control, C‐REV, 2′3′‐cGAMP and C‐REV+ 2′3′‐cGAMP. All groups have an equal average tumor size. C‐REV (1 × 10^6^ PFU per 50 μL PBS) was injected. The following day, 20 μg of 2′3′‐cGAMP in 50 μL PBS was injected. As for the control group, mice were injected with 50 μL saline solution. Clinical signs, bodyweight changes and tumor growth were monitored. Tumor volume was measured twice weekly until study termination. Tumor volume (*V*) was estimated using the equation: *V* = *L* × *W* ^2^/2, where *L* and *W* are tumor length and width, respectively.

### 2.2 Cell lines

Mouse squamous cell carcinoma cells (SCC‐VII cells; RRID: CVCL_V412) were gifted by Dr Masunaga (Kyoto University, Kyoto, Japan) and murine pancreatic ductal adenocarcinoma cells–Pan02 (RRID: CVCL_D627) and KPC (RRID: CVCL_XD12)–were kindly provided by Dr Sho (Nara Medical University) and Dr Ohuchida (Kyushu University), respectively. JAWSII cells (RRID: CVCL_3727, CRL‐3612) were purchased from ATCC (Manassas, VA, USA). African green monkey kidney cells (Vero cells; RRID: CVCL_0059) and PLAT‐E cells (RRID: CVCL_B488) were obtained from the RIKEN Cell Bank (Tsukuba, Japan) and Cell Biolabs Inc. (San Diego, CA, USA), respectively. ATCC has evaluated the authenticity of all cell lines within the past 3 years by analyzing their STR profiles. However, no registered STR profile corresponds to these cell lines within the ATCC database. The KPC cell line used in this study was independently established by Dr Ohuchida through tumors generated by KPC mice [29]. Consequently, the KPC cell line used in this study could have a STR profile that varies from what might be available commercially. The STR profiles of all three murine cell lines used in this study can be found in Table S1. All cell lines have been confirmed to be mycoplasma‐free through PCR (6601 TaKaRa PCR Mycoplasma Detection Set).

SCC‐VII cells, Pan02 cells, KPC cells and Vero cells were cultured in Dulbecco's modified Eagle medium with high glucose (DMEM; Wako, Osaka, Japan) and supplemented with 10% heat‐inactivated fetal bovine serum (FBS; F7524, Sigma, Tokyo, Japan), 100 IU·mL^−1^ penicillin, and 100 μg·mL^−1^ streptomycin (PS) (Wako). JAWSII cells were cultured in RPMI 1640 medium with 10% heat‐inactivated FBS, PS, 1 mm sodium pyruvate (Thermo Fisher Scientific, Tokyo, Japan), 0.1 mm nonessential amino acids (Thermo Fisher Scientific), and 10 ng·mL^−1^ mGM‐CSF (BioLegend, Tokyo, Japan). All the cells were cultured at 37 °C in a 5% CO_2_ humidified atmosphere. To establish the KPC cells that stably express human STING, human STING was cloned into pMX‐IRES‐GFP and transfected into PLAT‐E cells (Cell Biolabs, Inc.), packaging cells for retrovirus. KPC cells were transduced by culturing with retrovirus‐containing conditioned medium from PLAT‐E cells.

### 2.3 Viruses

C‐REV is an attenuated mutant clone derived from HSV‐1 strain HF. The virus was propagated in Vero cells and stored in aliquots at −80 °C. C‐REV was diluted in PBS for *in vivo* and *in vitro* experiments. Viral titers were assayed in Vero cells and are expressed as plaque‐forming units per milliliter (PFU·mL^−1^). C‐REV‐GM‐CSF was generated through the homologous recombination method wherein the targeting vector was inserted with the CMV‐murine GM‐CSF‐IRES‐ZsGreen (Clontech, Kusatsu, Shiga, Japan) sequence flanked by sequences from the UL42‐UL43 region of C‐REV genome.

### 2.4 Cell proliferation assay

Cell proliferation was determined using the 3‐(4,5‐dimethylthiazol‐2‐yl)‐2,5‐diphenyl tetrazolium bromide [MTT] dye reduction method. SCC‐VII cells, Pan02 cells and KPC cells were seeded, grown in DMEM, and incubated at 37 °C with 5% CO_2_. Cells were infected with C‐REV at several multiplicities of infection (MOI). After incubation for 1 h, DMXAA was added to the medium with respective doses. Viable cells were quantified by colorimetric MTT assay.

### 2.5 2′3′‐cGAMP treatment for cultured cells

Cells were seeded in DMEM with 10% FBS. The medium was then replaced with a buffer containing 50 mm Hepes (pH 7.4), 100 mm KCl, 3 mm MgCl_2_, 10 μg·mL^−1^ digitonin, 0.1 mm DTT, 85 mm sucrose, 0.2% BSA, 1 mm ATP, and 2′3′‐cGAMP. Cells were incubated for 30 min at 37 °C with 5% CO_2_. After which, the buffer was replaced with DMEM medium and incubated for 30 min at 37 °C with 5% CO_2_. After incubation, cells were corrected for western blotting.

### 2.6 Western blotting

Cells were lysed in 1× sample buffer (20 mm Hepes pH 7.3, 25 mm 2‐glycerophosphate, 50 mm NaCl, 1.5 mm MgCl_2_, 2 mm EDTA, 2% SDS, 5% β‐ME) and boiled for 10 min. The cell lysate was then subjected to immunoblotting with anti‐phospho‐TBK1 (CST, Tokyo, Japan, 5483), anti‐TBK1 (CST, 3504), anti‐phospho‐IRF3 (CST, 4947), anti‐IRF3 (Biolegend, clone 12A4A35), anti‐β‐actin (Santa Cruz, Dallas, TX, USA, sc‐81178) antibodies.

### 2.7 qPCR

Total RNA (0.5 μg) was isolated with Trizol reagent (Invitrogen, Tokyo, Japan) and reverse‐transcribed to generate cDNA using ReverTra Ace (TOYOBO, Osaka, Japan) for 1 h at 42 °C. The resulting cDNA was used as a template for the qRT‐PCR quantification of IFN‐β using THUNDERBIRD SYBR qPCR Mix (TOYOBO). The primer sequences used in this study are below. Mouse 18S fw: 5′‐CTTAGAGGGACAAGTGGCG‐3′, mouse 18S rev: 5′‐ACGCTGAGCCAGTCAGTGTA‐3′, mouse IFN‐β fw: 5′‐TCCAAGAAAGGACGAACATTCG‐3′, mouse IFN‐β rev: 5′‐TGCGGACATCTCCCACGTCAA‐3′. Quantification was performed on a StepOne Real‐time PCR system (Thermo Fisher Scientific) for cDNA amplification under the following conditions: 95 °C for 10 min, followed by 40 cycles of 95 °C for 30 s, and 60 °C for 1 min. Relative mRNA levels were determined using the comparative *C*_t_ method and normalized against 18S rRNA.

### 2.8 Flow cytometry analysis

Tumors were harvested on the indicated days, minced into small pieces, and enzymatically digested for 60 min at 37 °C with gentle agitation. For SCCVII tumors, the digestion buffer consists of collagenase IV (2 mg·mL^−1^, Worthington Biochemical Corporation, Lakewood, NJ, USA) and deoxyribonuclease I (100 μg·mL^−1^, Roche, Tokyo, Japan) in DMEM containing 5% FBS. For Pan02 and KPC tumors, collagen II (1 mg·mL^−1^, Worthington Biochemical Corporation) was added to the digestion buffer above. Single‐cell suspensions were obtained by gently passing the enzyme‐treated tumor through a 70‐μm cell strainer. The cells were then washed with PBS containing 5% FBS and 0.1% sodium azide for downstream analysis. Finally, cells were stained with the following mouse antibodies (BioLegend): CD45 Brilliant Violet 510, CD3 FITC, CD3 Brilliant Violet 421, CD8a FITC, CD8a APC‐Cy7, CD4 FITC, PD‐1 PerCP/Cy5.5, TIM3 APC, Ly6c FITC, Ly6c PerCP/Cy5.5, NKp46 PE, CD11b Brilliant Violet 421, CD11c APC‐Cy7, CD103 APC, I‐A/I‐E FITC, I‐A/I‐E PerCP/Cy5.5, F4/80 PE, KLRG1 PE, XCR1 PE, CD44‐biotin and streptavidin PerCP/Cy5.5.

### 2.9 TDLN lymphocytes for cytotoxicity assay

The tumor‐draining lymph nodes were harvested, passed through a 70‐mm strainer, and incubated with the ACK lysing Buffer (Gibco, Tokyo, Japan) to remove erythrocytes. Lymphocytes were cultured in RPMI with 10% FBS and interleukin2 (IL2; 100 U·mL^−1^) for 48 h at 37 °C and 5% CO_2_. Subsequently, lymphocytes were seeded in 24‐well plates pre‐seeded with KPC cells per the indicated ET ratios. The KPC cells used were pretreated with IFN‐α (25 ng·mL^−1^) for 30 min and then detached with trypsin–EDTA before being seeded on the 24‐well plates. After co‐culturing the lymphocytes and KPC cells for 24 h, tumor cell cytotoxicity was measured using an LDH assay (Cytotoxicity LDH assay kit‐WST, Dojindo, Tokyo, Japan) using the medium from the co‐culture. Blank LDH levels were subtracted from the LDH values of each sample, and results were normalized by the highest value in the assay.

### 2.10 TDLN lymphocytes for ELISPOT assay

We used the tumor‐bearing mice (control) and mice that showed complete response from the rechallenge experiments in Fig. 5C. Forty‐eight hours before taking the tumor‐draining lymph nodes, mice were subcutaneously injected with 1 × 10^5^ KPC cells pretreated with IFN‐α (25 ng·mL^−1^) for 30 min before detachment. Lymphocytes from the tumor‐draining lymph nodes were cocultured with tumor cells in the same way as the cytotoxicity assay. After 24 h, cells were detached and centrifuged to separate the cells from the medium that was used for the LDH cytotoxicity assay as described earlier. Thereafter, 1 × 10^5^ cells from the centrifuged cells were subjected to another 12‐h culture on a 96‐well plate precoated by anti‐IFN‐γ antibodies for the ELISPOT assay (U‐CyTech, Utrecht, Netherlands).

### 2.11 Statistical analysis

Statistical comparisons were performed using the prism software, version 9.1.2 (GraphPad Software, Boston, MA, USA). Statistical significance between the two groups was analyzed using an unpaired Student's *t*‐test. Two‐way analysis of variance (ANOVA) with Dunnett's or Tukey's post‐test was used for experiments involving three groups and/or analysis of multiple time points. *P*‐values < 0.05 were considered to be statistically significant.

## 3 Results

### 3.1 Tumor cells activate their STING pathway only to a limited extent

**Fig. 1:**
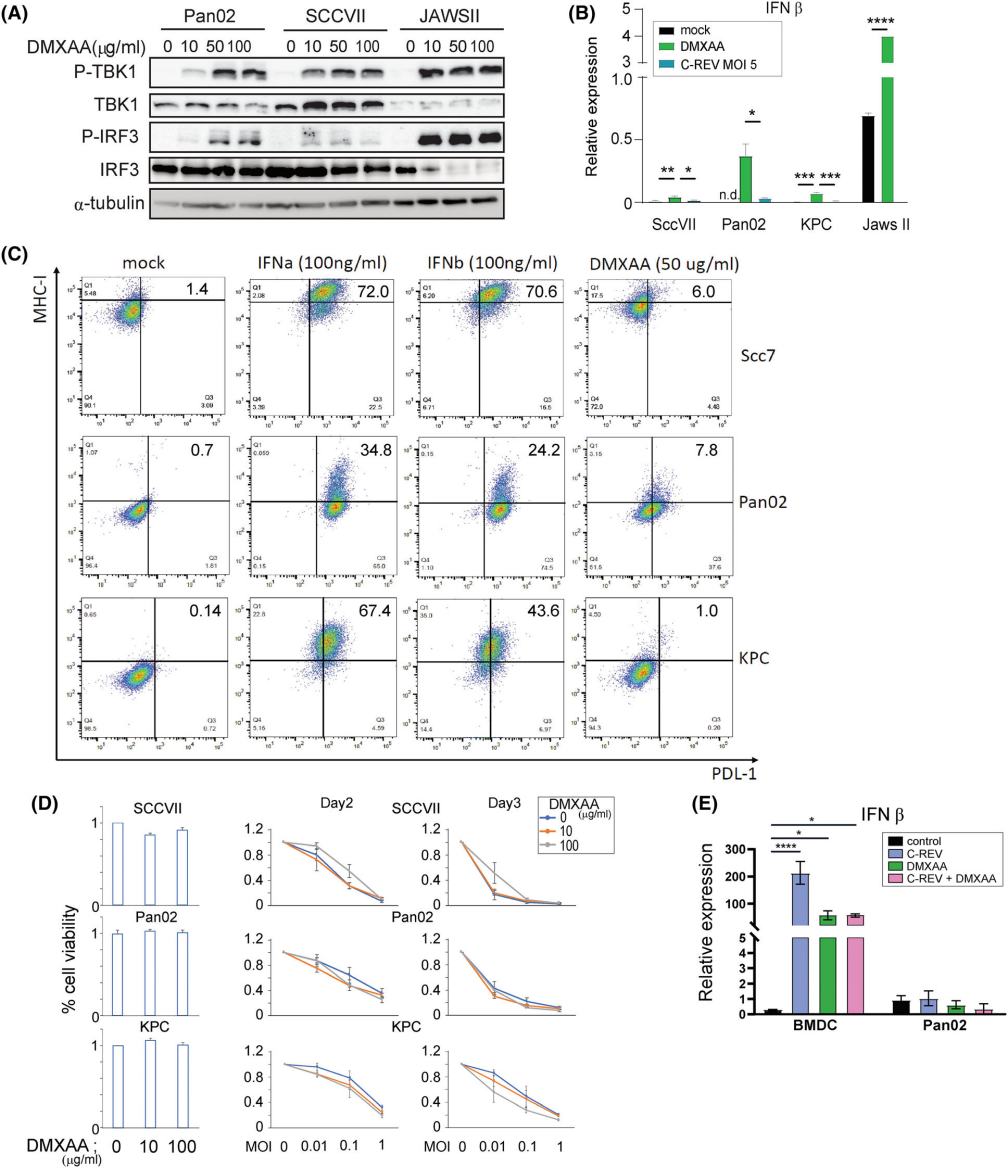
The murine cancer cells have a defective STING pathway, which induces type I IFN inefficiently. (A) Western blot analysis on the indicated proteins of the STING pathway. At 1 h post‐infection with C‐REV at MOI 5, cells were treated with the indicated DMXAA concentrations (0, 50, 10 or 100 μg·mL^−1^), and at 4 hpi, cells were subjected to western blot analysis. The data presented are representative of three independent experiments. (B) Induction of mIFN‐β. Respective cells were treated with DMXAA (50 μg·mL^−1^) or C‐REV. After 6 h, cells were subjected to RNA extraction and analyzed by RT‐qPCR. mIFN‐β expression was normalized relative to the expression of the 18S rRNA. Data are presented as mean ± SEM (*n* = 3). Tukey's multiple comparison tests were performed on the three samples from each cell line. Student's *t*‐test was performed between the two samples from JAWSII cells. n.d., not detected. (C) Representative results of the flow analysis of MHC class‐I and PD‐L1 expression level in SCCVII, Pan02 and KPC cells 12 h after the indicated treatment. Three independent experiments were performed. (D) Cytotoxicity of C‐REV at the indicated MOI against SCCVII, Pan02 and KPC cells on Day2 and Day3 by MTT assay. Cells were treated with DMXAA at 1 hpi. Data are presented as mean ± SEM (*n* = 3). (E) Induction of mIFN‐β in bone marrow‐derived dendritic cells (BMDC) or Pan02 cells after treatment of C‐REV and/or DMXAA. Cells were treated with DMXAA (50 μg·mL^−1^) or C‐REV (MOI 5). Cells were then subjected to RNA extraction at 6 hpi and analyzed by RT‐qPCR. mIFN‐β expression was normalized relative to the expression of the 18S rRNA. Data are presented as mean ± SEM (*n* = 3). One‐way ANOVA followed by Dunnett's multiple comparison tests were performed. *P < 0.05, **P < 0.01, ***P < 0.001, ****P < 0.0001.

To assess the feasibility of the combination therapy of C‐REV, an OV, and 2′3′‐cGAMP, an endogenous membrane‐impermeable STING agonist, we first characterized the integrity of the STING pathway in murine tumor cell lines SCCVII, Pan02 and KPC. We stimulated SCCVII cells and Pan02 cells using several doses of DMXAA, a well‐known membrane‐permeable STING agonist. For both SCCVII cells and Pan02 cells, the lowest dose of DMXAA, 10 μg·mL^−1^, already induced limited but noticeable levels of P‐TBK1, the direct downstream agent of an activated STING molecule (Pan02 and SCCVII, Fig. 1A). Despite such P‐TBK1 levels, its downstream target, phosphorylated IRF‐3 (P‐IRF3), which is crucial for the expression of genes responsible for antiviral defense, was present only in negligible levels. Although the level of phosphorylated TBK1 in both cell lines increased proportionate to the DMXAA dose, an increase in the level of P‐IRF3 was observed only in Pan02 cells.

To compare the level of responsiveness of the tumoral STING pathway with non‐tumor cells, a murine DC cell line, JAWS‐II, was used. In contrast to the SCCVII and Pan02 cells, JAWSII cells displayed a fully activated STING pathway even when only the lowest DMXAA dose was used, as indicated by the significant levels of P‐TBK1 and P‐IRF3 (JAWSII, Fig. 1A), suggesting that the activation of the STING pathway in both tumor cells occurred only to a much lesser extent than in non‐malignant cells. Note that the anti‐IRF3 antibody used was manufactured to recognize only the non‐phosphorylated form of IRF3. Since most of the IRF3 has already been converted to P‐IRF3 in JAWSII cells, anti‐IRF3 detected only a limited IRF3 amount, whereas the opposite was observed for both tumor cell lines (Pan02 and SCCVII, Fig. 1A).

To confirm the defectiveness of the tumoral STING pathway, we also examined the IFN‐β mRNA transcription level by RT‐qPCR after STING agonist treatment. Consistent with the western‐blotting results, all three murine tumor cell lines, especially Pan02 cells, managed to express IFN‐β upon addition of a high dose of DMXAA treatment (50 μg·mL^−1^) but at a much lower level relative to JAWSII cells (Fig. 1B). The KPC cell line, derived from induced pancreatic ductal adenocarcinoma in KPC mice (LSL‐KrasG12D/+; LSL‐Trp53R172H/+; Pdx1‐Cre), is well regarded as a faithful representation of human pancreatic cancer. Similar to SCCVII cells, KPC cells failed to induce IFN‐β mRNA, indicating that KPC also carries a highly defective STING pathway (KPC, Fig. 1B).

In line with this observed limited functionality of the STING pathway in KPC, Pan02, and SCCVII, DMXAA treatment also failed to increase the surface expression of PDL‐1 and MHC‐I significantly, genes reported to be upregulated upon treatment with type I IFN (Fig. 1C). Although an increase in the expression of PDL‐1 and MHC‐I was observed in Pan02 cells, such increase is negligible compared with the increase in expression of PDL‐1 and MHC‐I observed after either IFN‐α or IFN‐β treatment (Fig. 1C). The failure of the three tumor cell lines to respond significantly upon DMXAA treatment can be attributed to a lack of type I IFN, suggesting that even with a permeable STING agonist, the extensive defects in their STING pathway rendered the tumor cell lines incapable of expressing significant quantities of type I IFN.

### 3.2 STING agonists exert negligible effects on C‐REV replication in tumor cells

We have previously reported that C‐REV retains high infectious ability against various human pancreatic tumor cell lines even in the presence of 2′3′‐cGAMP and digitonin [28]. C‐REV infection also only induced a minimal level of IFN‐β expression in all three tumor cell lines (Fig. 1B), demonstrating that the tumoral STING pathway, a cellular antiviral mechanism, failed to respond significantly to a viral infection. To examine further whether the STING activation in KPC, SCCVII and Pan02 cells affects the cytotoxicity of C‐REV, we infected the cells with C‐REV at multiplicities of infection (MOI) 0.01, 0.1, 1 or 10 followed by adding DMXAA 1 h post‐infection (1 hpi). A high dose of DMXAA alone exerted no effect on the viability of the three murine tumor cell lines (left, Fig. 1D). Consistent with our previous reports, C‐REV retained high cytotoxicity against all three tumor cell lines even in the presence of DMXAA (right, Fig. 1D). Increasing the C‐REV MOI proportionately decreased the tumor cellular viability and this trend was not significantly altered even when the DMXAA dose was increased 10‐fold. The minimal IFN‐β expression observed upon treating Pan02 cells with both C‐REV and 2′3′‐cGAMP further corroborates the inability of STING agonists to affect C‐REV replication in tumor cells (Fig. 1E). Compared with bone marrow‐derived DC, which exhibited significant IFN‐β expression upon addition of either C‐REV alone or with DMXAA, negligible differences were observed from Pan02 cells regardless of the treatment (Fig. 1E). Taken together, all the tumor cell lines possess a fragile STING pathway incapable of suppressing C‐REV replication.

### 3.3 None of the three tumor cell lines was able to uptake soluble 2′3′‐cGAMP

**Fig. 2:**
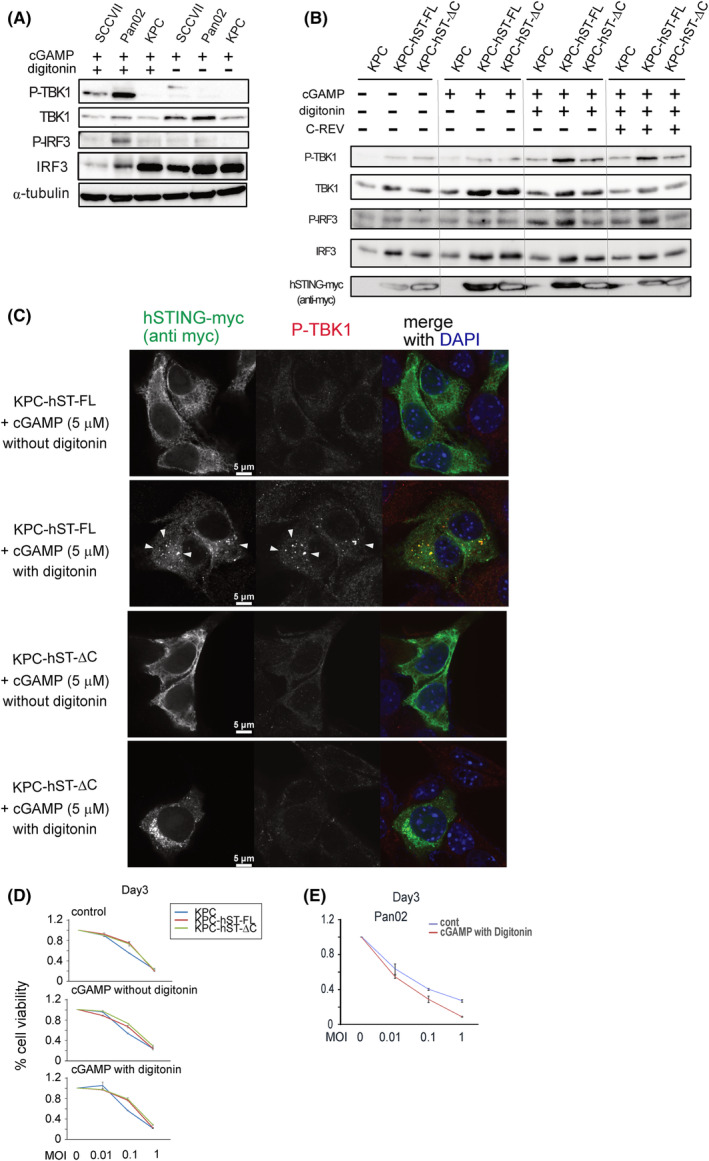
The murine cancer cells were unable to uptake 2′3′‐cGAMP. (A,B) Western blot analysis on the indicated proteins on the STING pathway. Cells were lysed 3 h after adding 2′3′‐cGAMP (5 μm) with or without digitonin and then subjected to western blot analysis. The data presented are representative of three independent experiments. (A) SCCVII, Pan02 or KPC cells. (B) KPC cells or KPC cells that stably express human STING full‐length (hST‐FL) or human STING ΔC (hST‐ΔC) (a mutant which lacks 9 a.a. of C terminus). (C) The typical images of immunofluorescent cell staining. KPC hST‐FL cells or KPC hST‐ΔC cells were cultured on coverslips and treated with 2′3′‐cGAMP (5 μm) in the presence or absence of digitonin. After 3 h, cells were fixed with formaldehyde, permeabilized by MeOH, and stained with the indicated primary antibody. White arrows indicate the dots formed by the activation of the STING pathway. Bars represent 5 μm. (D,E) Cytotoxicity of C‐REV at the indicated MOI against KPC, KPC hST‐FL or KPC‐hST‐ΔC cells (D) or Pan02 cells (E) on Day 3 by MTT assay. At 1 hpi, 2′3′‐cGAMP (5 μm) was introduced in the presence or absence of digitonin. Data are presented as mean ± SEM (*n* = 3).

To confirm whether the tumor cells can uptake 2′3′‐cGAMP, it was added to cells with or without digitonin, a membrane permeabilizing agent. All three cancer cell lines, except for KPC, displayed noticeable levels of P‐IRF3 upon adding 2′3′‐cGAMP with digitonin. However, no detectable activation of the STING pathway was observed when 2′3′‐cGAMP was added alone, suggesting that those tumor cells could not uptake 2′3′‐cGAMP (Fig. 2A).

Consistent with the inability of KPC cells to induce IFN‐β after DMXAA treatment (KPC, Fig. 1B), KPC cells failed to activate the STING pathway even when 2′3′‐cGAMP was administered with digitonin, indicating that KPC cells carry a highly defective STING pathway. To restore the STING pathway of KPC cells, we introduced human STING full‐length into the KPC cells (KPC‐hST‐FL) and, as a control, introduced the dominant negative of human STING, i.e. STING lacking 9 amino acids in its C‐terminus (KPC‐hST‐ΔC) [30]. As expected, both KPC‐hST‐FL cells and KPC‐hST‐ΔC cells were still unable to uptake 2′3′‐cGAMP (Fig. 2B). However, introducing the full‐length human STING gene into the KPC cells partially restored the STING pathway of KPC cells, since a notable STING pathway activation was observed from KPC‐hST‐FL cells after being treated with 2′3′‐cGAMP and digitonin while KPC cells and KPC‐hST‐ΔC cells remained unresponsive (Fig. 2B). Immunofluorescent cell staining also showed that 2′3′‐cGAMP and digitonin treatment successfully induced the translocation of the introduced human STING‐FL to the Golgi area and that the characteristic dot‐like clustered structures of human STING‐FL match the localization of P‐TBK1 (KPC‐hST‐FL + cGAMP with digitonin; Fig. 2C). In contrast, although STING‐ΔC also translocated to the Golgi area after 2′3′‐cGAMP and digitonin treatment, no P‐TBK1 signal was detected in KPC‐hST‐ΔC cells (KPC‐hST‐ΔC + cGAMP with digitonin; Fig. 2C). These results suggest that regardless of the integrity of the tumoral STING pathway, tumor cells are unable to uptake 2′3′‐cGAMP.

We also examined whether C‐REV can suppress the restored STING pathway in KPC‐hST‐FL cells. Although western blotting results indicate that C‐REV was unable to inhibit TBK1 and IRF3 activation when cells were treated with 2′3′‐cGAMP and digitonin (Fig. 2B), 2′3′‐cGAMP influx failed to alter C‐REV cytotoxicity against KPC cells and KPC‐hST‐FL cells (Fig. 2D). Similarly, 2′3′‐cGAMP influx also failed to decrease C‐REV cytotoxicity against Pan02 cells, which also carry a partially responsive STING pathway (Fig. 2E). Overall, both STING agonists, 2′3′‐cGAMP and DMXAA, exerted only a marginal effect on C‐REV cytotoxicity and all the tumor cell lines possessed a fragile STING pathway incapable of suppressing C‐REV replication.

### 3.4 Gamma 34.5 gene confers an inhibitory effect against partially responsive STING pathways

Since C‐REV is a naturally occurring attenuated virus, C‐REV retains a functional gamma 34.5 gene, whereas most other HSV‐1‐based OVs do not. The gamma 34.5 gene has been reported as one of the principal HSV‐1 genes involved in suppressing the STING pathway of the host cell [31]. This gene encodes ICP 34.5, which binds to STING and prevents its translocation from the ER to the Golgi compartment. In doing so, ICP34.5 prevents STING from interacting with TBK1 and other downstream effectors necessary for stimulating the expression of Type I IFN.

To confirm that ICP34.5 is a factor for C‐REV suppressive ability against STING, we evaluated the degree to which the STING pathway is activated when tumor cells are infected with HSV‐1, C‐REV or other HSV‐1‐based OVs lacking a functional gamma 34.5 gene. R3616, an OV lacking gamma 34.5, failed to inhibit STING translocation and TBK1 activation (Fig. S1A). Cells infected with hrR3 and C‐REV, viruses that contain a functional gamma 34.5 gene, demonstrated partial inhibition of STING translocation and TBK1 activation. Consistently, cells infected with wild‐type HSV‐1 demonstrated complete inhibition of STING translocation and TBK1 activation. These results indicate that a functional gamma 34.5 gene could have contributed to C‐REV inhibitory effect against the STING pathway. Given that C‐REV can suppress a partially responsive STING pathway during 2′3′‐cGAMP influx, the inability of tumor cells to uptake 2′3′‐cGAMP further ensures that 2′3′‐cGAMP cannot hamper C‐REV tumoral replication.

### 3.5 Combination therapy enhances antitumor efficacy

**Fig. 3:**
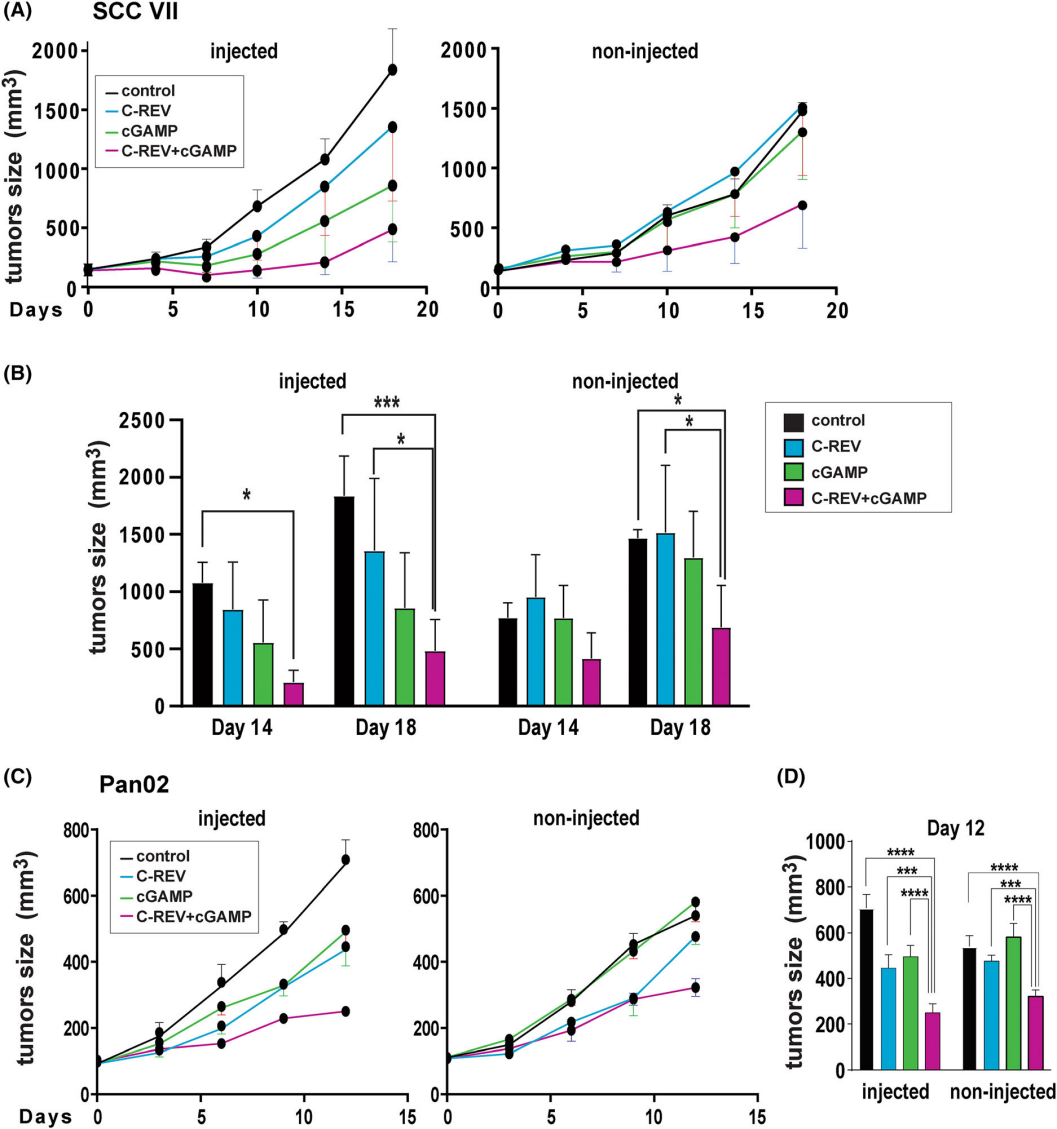
The combination therapy enhanced the abscopal effect in the immunocompetent tumor model. (A) Tumor growth curves in SCCVII tumor model. Tumors were inoculated in C3H mice. C‐REV (1 × 10^6^ pfu) was injected into one tumor (IT) on Day 0. 2′3′‐cGAMP (20 μg) was intratumorally injected on Day 3 and Day 6 to the same tumor treated on Day 0. Data are presented as mean ± SEM (*n* = 4 mice). (B) Tumor size from (A) on Day 14 and Day 18 from both flanks. Two‐way ANOVA followed by Dunnett's multiple comparison tests were performed. *P < 0.05, ***P < 0.001. (C) Tumor growth curves in Pan02 tumor model. Tumors were inoculated in B6 mice. One of the tumors was treated with C‐REV (1 × 10^6^ pfu) and 2′3′‐cGAMP (20 μg) following the same schedule as in (A). Data are presented as mean ± SEM (*n* = 5 mice). (D) Tumor size from (C) on Day 12 from both flanks. Two‐way ANOVA followed by Dunnett's multiple comparison tests were performed. *P < 0.05, ***P < 0.001.

Previous studies have reported that intratumoral injection of 2′3′‐cGAMP exerts a significant antitumor effect primarily due to the ability of 2′3′‐cGAMP to induce DC activation upon DC uptake [32, 33]. From this, we hypothesized that 2′3′‐cGAMP treatments can work synergistically with C‐REV *in vivo*, resulting in greater antitumor efficacy. To this end, we assessed the tumor growth rate in a bilateral SCC‐VII tumor model wherein mice were subcutaneously inoculated with SCC‐VII tumors in both flanks. C‐REV was intratumorally injected into one of the tumors on day 0, thereaftreferred to as the injected side, followed by intratumoral injection of 2′3′‐cGAMP on days 3 and days 6 on the same side. As expected, combination therapy showed better antitumor efficacy on both injected and non‐injected sides by day 18 (Fig. 3A) than did C‐REV monotherapy (Fig. 3B), suggesting that combination therapy not only improved immune responses against tumor cells on the injected side but also enhanced the abscopal antitumor effects on the non‐injected side. Similarly, in the bilateral Pan02 tumor model, combination therapy significantly suppressed the tumor growth in both flanks (Fig. 3C,D). Synergistic effect calculations on the tumor volumes also revealed that 2′3′‐cGAMP had not only an additive effect but also a synergistic effect with C‐REV's own antitumor functions (Table 1).

**Table 1:** Fractional tumor volume (FTV) after treatment with C‐REV, either alone or in combination with 2′3′‐cGAMP. E‐FTV, expected FTV (mean FTV of C‐REV) × (mean FTV of 2′3′‐cGAMP or ADU‐S100); FTV, fractional tumor volume (mean tumor volume experimental/mean tumor volume control); O‐FTV, observed FTV. Synergic effect: E‐FTV/O‐TV > 1 in red.

SccVII											
Day	Injected					Day	Non‐injected				
	FTV	FTV	E‐FTV	O‐FTV	E‐FTV/O‐FTV		FTV	FTV	E‐FTV	O‐FTV	E‐FTV/O‐FTV
	CREV	cGAMP	comb.	comb.	comb.		CREV	cGAMP	comb.	comb.	comb.
0	0.998	0.972	0.971	0.932	1.041	0	1.139	1.056	1.203	1.008	1.193
3	0.913	1.003	0.916	0.668	**1.372**	3	1.140	1.345	1.533	0.948	**1.618**
6	0.544	0.767	0.417	0.300	**1.391**	6	1.034	1.217	1.259	0.748	**1.684**
9	0.407	0.629	0.256	0.210	**1.222**	9	0.941	1.062	1.000	0.517	**1.932**
12	0.515	0.785	0.404	0.195	**2.077**	12	0.998	1.232	1.230	0.545	**2.255**
18	0.467	0.740	0.345	0.264	**1.309**	18	0.886	1.034	0.917	0.473	**1.938**

Pan02											
Day	Injected					Day	Non‐injected				
	FTV	FTV	E‐FTV	O‐FTV	E‐FTV/O‐FTV		FTV	FTV	E‐FTV	O‐FTV	E‐FTV/O‐FTV
	CREV	cGAMP	comb.	comb.	comb.		CREV	cGAMP	comb.	comb.	comb.
0	0.976	0.941	0.918	0.941	0.976	0	0.972	1.005	0.976	0.968	1.009
3	0.716	0.839	0.600	0.732	0.820	3	0.821	1.109	0.911	0.923	0.987
6	0.611	0.784	0.479	0.452	1.060	6	0.779	1.028	0.801	0.689	1.162
9	0.667	0.668	0.446	0.459	0.971	9	0.642	0.954	0.612	0.634	0.965
12	0.630	0.700	0.441	0.353	**1.251**	12	0.884	1.077	0.952	0.596	**1.596**

KPC											
Day	Injected					Day	Non‐injected				
	FTV	FTV	E‐FTV	O‐FTV	E‐FTV/O‐FTV		FTV	FTV	E‐FTV	O‐FTV	E‐FTV/O‐FTV
	CREV	cGAMP	comb.	comb.	comb.		CREV	cGAMP	comb.	comb.	comb.
0	0.810	0.559	0.453	0.810	0.559	0	0.754	0.837	0.631	0.892	0.707
4	0.576	0.508	0.293	0.296	0.988	4	0.570	0.629	0.358	0.454	0.789
7	0.478	0.329	0.157	0.191	0.823	7	0.598	0.884	0.529	0.488	1.084
11	0.364	0.386	0.141	0.155	0.906	11	0.828	1.140	0.944	0.723	**1.306**
14	0.388	0.390	0.152	0.147	1.030	14	0.759	0.939	0.713	0.627	**1.138**
18	0.509	0.423	0.215	0.148	**1.459**	18	0.695	0.838	0.582	0.493	**1.180**

SccVII											
Day	Injected					Day	Non‐injected				
	FTV	FTV	E‐FTV	O‐FTV	E‐FTV/O‐FTV		FTV	FTV	E‐FTV	O‐FTV	E‐FTV/O‐FTV
	C‐REV	ADU‐S100	comb.	comb.	comb.		C‐REV	ADU‐S100	comb.	comb.	comb.
0	1.005	0.973	0.978	1.001	0.977	0	0.979	0.979	0.958	0.979	0.979
3	0.572	0.857	0.490	0.833	0.588	3	0.765	0.940	0.719	0.999	0.719
7	0.482	0.412	0.199	0.254	0.781	7	0.791	0.792	0.626	0.712	0.879
10	0.395	0.319	0.126	0.119	1.060	10	0.709	0.670	0.475	0.728	0.652
14	0.373	0.226	0.084	0.080	1.054	14	0.926	0.666	0.617	0.606	1.017
17	0.397	0.247	0.098	0.060	**1.643**	17	0.853	0.624	0.532	0.586	0.908
21	0.337	0.227	0.077	0.061	**1.264**	21	0.753	0.611	0.461	0.514	0.896

To examine whether other STING agonists can also act with C‐REV synergistically, we examined the combination therapy of C‐REV with ADU‐S100, a known STING agonist currently undergoing clinical trial [34]. Even with a low dose of ADU‐S100 (8 μg) in the SCC‐VII tumor model, the combination therapy showed the most substantial antitumor effects among the groups (Fig. S2A). However, the abscopal effects on the contralateral side were less than that of the combination treatment of C‐REV with 2′3′‐cGAMP (Fig. S2A, Table 1). These results suggest that even across different types of tumor models, the combination therapy of C‐REV with STING agonists, especially 2′3′‐cGAMP, could enhance the systemic antitumor immunity elicited by C‐REV.

### 3.6 Combination therapy efficiently induces an increasing number of infiltrated lymphocytes

**Fig. 4:**
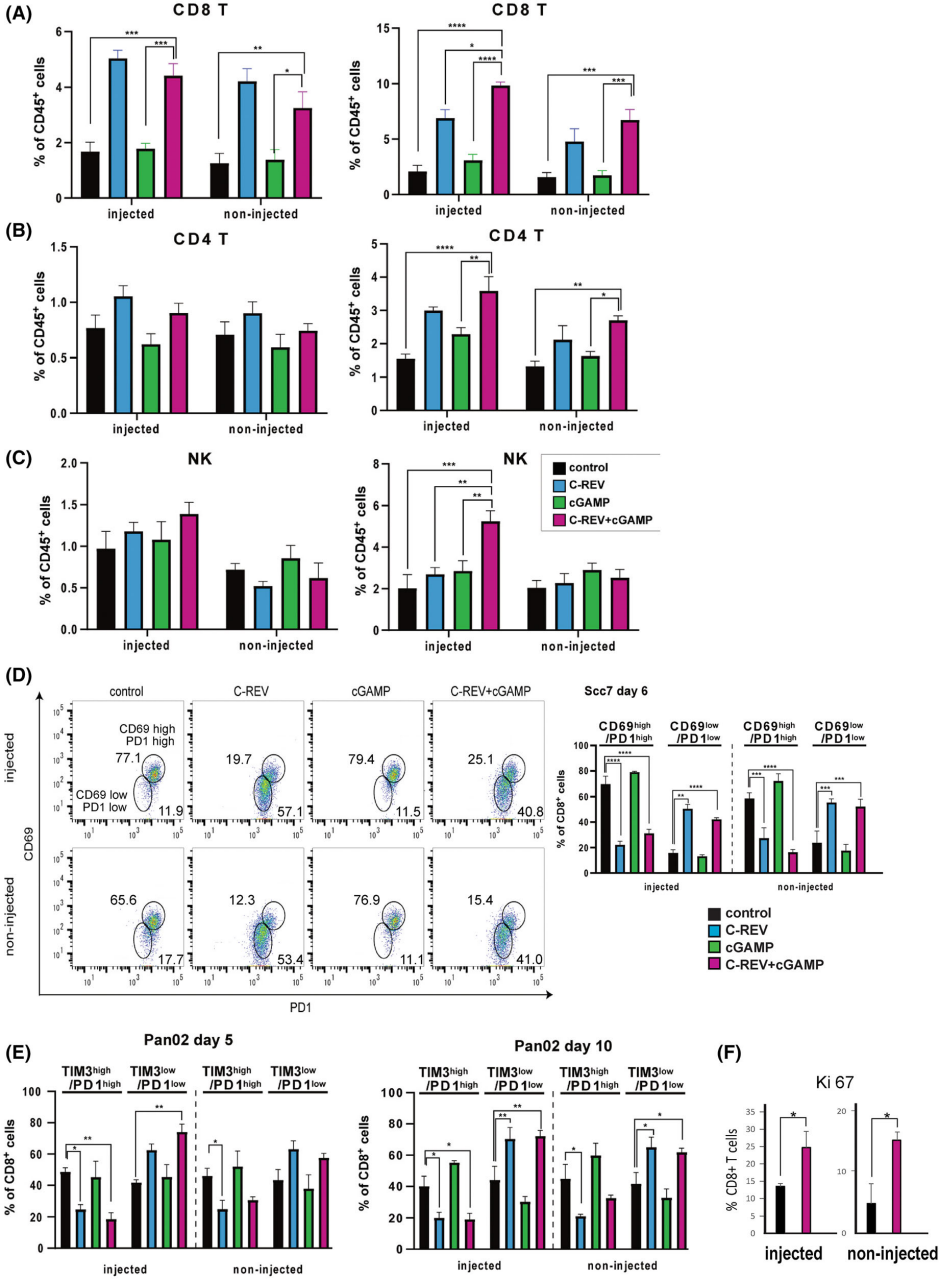
The combination therapy enhanced the tumoral recruitment of CD8^+^ T‐cells. (A–C) Flow analysis of the tumor‐infiltrated lymphocytes (TIL). SCCVII tumors were harvested on Day 6, digested enzymatically and stained for surface markers. CD4^+^ T‐cells were selected as CD45^+^CD3^+^CD4^+^ cells. CD8^+^ T‐cells were selected as CD45^+^CD3^+^CD8^+^ cells. NK cells were selected as CD45^+^CD11b^−^CD3^−^NKp46^+^ cells. Data are presented as mean ± SEM (*n* = 3 mice). One‐way ANOVA followed by Dunnett's multiple comparison tests were performed. (D) Dot charts of CD8^+^ T‐cells from SCCVII tumors were stained with CD69 and PD1. Bar graphs were presented as mean ± SEM (*n* = 3 mice). Student's *t*‐tests were performed. *P < 0.05, **P < 0.01, ***P < 0.001, ****P < 0.0001. (E) Flow analysis of the tumor‐infiltrated lymphocytes from the Pan02 tumors harvested on the indicated day. CD8^+^ T‐cells were stained with TIM3 and PD1. Data are presented as mean ± SEM (*n* = 3 mice). One‐way ANOVA followed by Dunnett's multiple comparison tests were performed. (F) Flow analysis of the Ki67 expression level on the CD8^+^ T‐cells from SCCVII tumors on Day 6. Data are presented as mean ± SEM (*n* = 3 mice). Student's *t*‐tests were performed. *P < 0.05, **P < 0.01, ***P < 0.001, ****P < 0.0001.

To gain insights into how the combination therapy enhanced C‐REV antitumor efficacy, we examined different populations of tumor‐infiltrating lymphocytes (TIL) from treated SCC‐VII tumors 6 and 12 days after the initial treatment. Mice treated with the combination therapy had higher percentages of tumor‐infiltrating CD8^+^ T‐cells and CD4^+^ T‐cells in both injected and non‐injected tumors compared with mice treated with either control or 2′3′‐cGAMP (Fig. 4A,B). On day 6, the percentages of CD8^+^ T‐cells and CD4^+^ T‐cells were comparable between the C‐REV‐treated and the combination therapy groups. However, by day 12, the combination therapy group had already displayed higher percentages of CD8^+^ T‐cells and CD4^+^ T‐cells in both flanks. Aside from increasing CD8^+^ T‐cells and CD4^+^ T‐cells, the combination therapy had already started displaying the highest number of tumor‐infiltrating NK cells by day 6, clearly exhibiting this trend by day 12 (Fig. 4C). OV infection is known to attract natural killer (NK) cells, as these cells assist in clearing viral infections. Considering that OV infection typically lasts only for a few days, the ability of combination therapy to sustain the increased number of NK cells until day 12 might imply that the increase in tumor‐infiltrated CD8^+^ and CD4^+^ T‐cells corresponded to an increase in the cytokines secreted by CD8^+^ and CD4^+^ T‐cells that assists in maintaining NK cells.

We then characterized the tumor‐infiltrating CD8^+^ T‐cells across the SCC‐VII tumor treatment groups. CD8^+^ cells from both the C‐REV monotherapy and the combination therapy expressed PD1 and CD69 lowly. Meanwhile, CD8^+^ T‐cells from the 2′3′‐cGAMP monotherapy shared the same features as that of control – high PD1 and high CD69 (Fig. 4D). A greater number of Ki67‐positive proliferative CD8^+^ T‐cells are also present in the combination therapy‐treated group than in the control group. A similar trend in CD8^+^ T‐cell characteristics was observed among treated Pan02 tumors. On both days 5 and 10, the infiltrated CD8^+^ T‐cells from the C‐REV‐treated or combination‐treated tumors expressed lower PD1 and lower TIM3 compared with CD8^+^ T‐cells from control or 2′3′‐cGAMP‐treated tumors (Fig. 4E). These observations suggest that the enhanced antitumor efficacy of combination therapy could be attributed to an increase in the number of infiltrated unexhausted CD8^+^ T‐cells and CD4^+^ T‐cells which can be sustained until later time points.

To understand the decrease in abscopal effects observed from the combination therapy of C‐REV with ADU‐S100, we also examined TIL on the SCC‐VII tumors treated with C‐REV and ADU‐S100. Although we observed enhanced antitumor effects in the combination of C‐REV with ADU‐S100, the percentage of CD45^+^ TIL in ADU‐S100‐treated tumors clearly decreased compared with control (Fig. S2B). Accordingly, the number of CD8^+^ T‐cells and CD4^+^ T‐cells in the combination therapy also decreased compared with the single treatment of C‐REV (Fig. S2B). This reduction of TIL could suggest that ADU‐S100 hyperactivated pre‐existing TIL and led them to apoptosis, thereby failing to activate systemic antitumor immunity.

### 3.7 Combination therapy confers sustained antitumor immunity

**Fig. 5:**
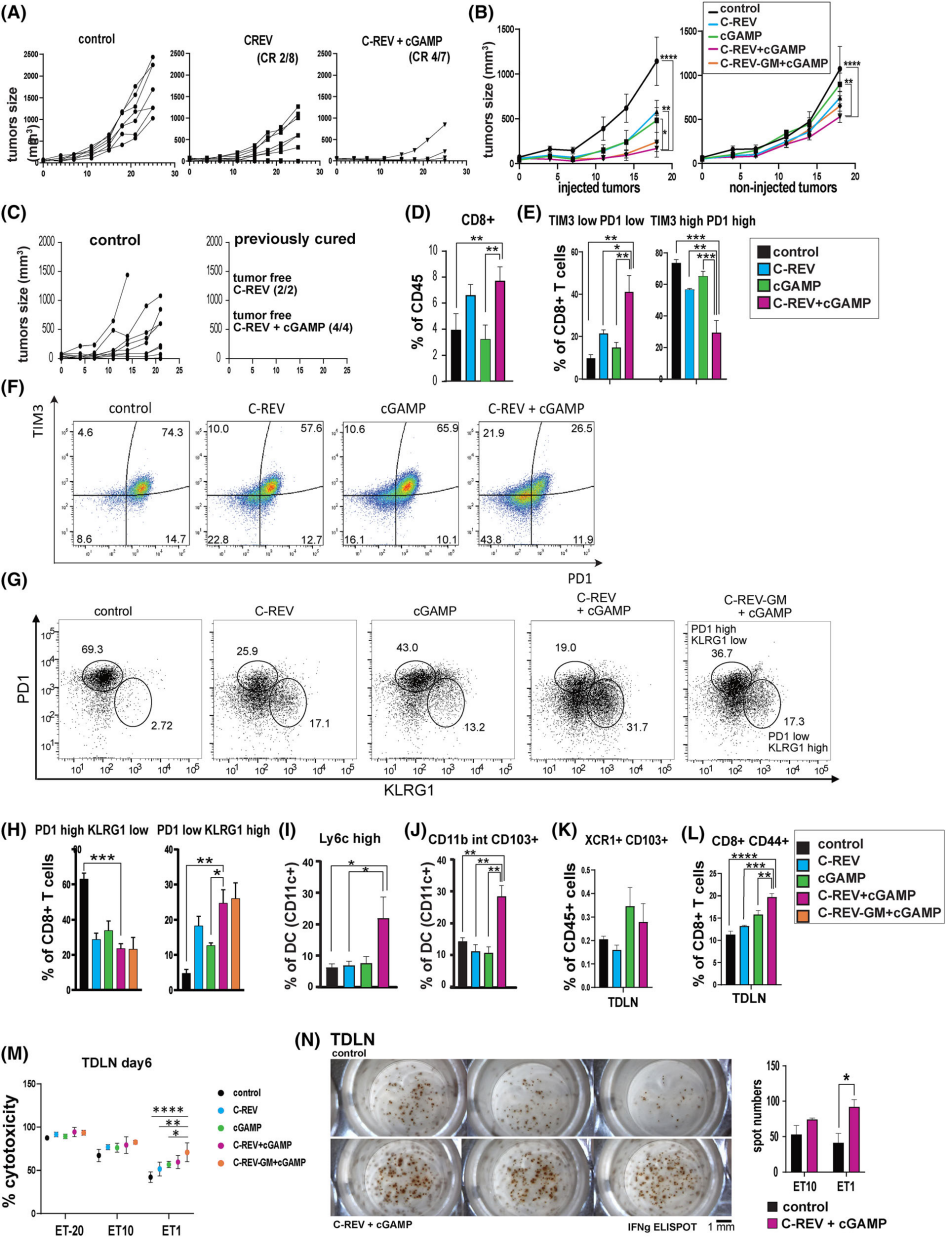
The combination therapy promoted the activation of antitumor‐specific CD8^+^ T‐cells. (A) Tumor growth curves of the KPC unilateral tumor model. Tumors were inoculated in B6 mice. C‐REV (2 × 10^6^ pfu) was injected into one tumor (IT) on Day 0 and Day 7. 2′3′‐cGAMP (15 μg) was intratumorally injected on Day 2, Day 4, Day 9 and Day 11 into the same tumor treated with the virus. Data are presented as mean ± SEM (*n* = 7 or 8 mice). (B) Tumor growth curves of KPC tumors on both flanks. C‐REV or C‐REV‐mGM‐CSF (1 × 10^6^ pfu) was injected into the one tumor (IT) on Day 0. 2′3′‐cGAMP (15 μg) was intratumorally injected on Day 3 and Day 6 into the same tumor treated on Day 0. Data are presented as mean ± SEM (*n* = 5 mice). Two‐way ANOVA followed by Dunnett's multiple comparison tests were performed. (C) Tumor growth curves of the KPC rechallenge experiment. Cultured KPC cells (3 × 10^6^ cells) were subcutaneously injected into the age‐matched naïve control mice (*n* = 10) and tumor‐free mice from (A) 1 month after CR was observed. (D,E) Flow analysis of the tumor‐infiltrated lymphocytes. KPC tumors were harvested on Day 8, digested enzymatically and stained for surface markers. CD8^+^ T‐cells were selected as CD45^+^CD3^+^CD8^+^ cells, and then analyzed by TIM3 and PD1. One‐way ANOVA followed by Dunnett's multiple comparison tests were performed. Data are presented as mean ± SEM (*n* = 3 mice). (F) Dot charts of CD8^+^ T‐cells from the KPC TIL analyzed in (E). (G) Dot charts of CD8^+^ T‐cells from KPC TIL analyzed on Day 6. CD45^+^CD3^+^CD8^+^ cells were analyzed by PD1 and KLRG1. (H) Bar graphs depicting flow analysis on KPC TIL in (G). Data are presented as mean ± SEM (*n* = 3 mice). One‐way ANOVA followed by Dunnett's multiple comparison tests were performed. (I–L) Bar graphs depicting the flow analysis of KPC TIL on Day 8 (I and J) or lymphocytes from tumor‐draining lymph node (TDLN) (K and L). DC cells were selected as CD45^+^CD11b^+^MHC‐classII highCD11c^+^ cells, and then analyzed by Ly6c (I) or CD103 (J). CD8^+^CD11c^+^ cells were analyzed by XCR1 and CD103 (K). CD3^+^CD8^+^ T‐cells were analyzed by CD44 (L). Data are presented as mean ± SEM (*n* = 3 mice). One‐way ANOVA followed by Dunnett's multiple comparison tests were performed. (M) Cytotoxicity assay of the TDLN lymphocytes against KPC cells during co‐culture. The TDLN were harvested on Day 6 from KPC tumor‐bearing mice. Whole lymphocytes from each TDLN were co‐cultured with KPC tumor cells with the indicated ET ratio in an IL2‐containing medium for 24 h. The co‐cultured medium was subjected to LDH measurement. Data are presented as mean ± SEM (*n* = 3 mice). Each ET ratio was performed with triplicates. Naïve‐wt mice without any history of tumor inoculation were used as a negative control. One‐way ANOVA followed by Dunnett's multiple comparison tests were performed. (N) Representative images of the ELISPOT assay for IFN‐γ (left) and spot numbers (right) from the co‐culture. Whole lymphocytes from the lymph node of the rechallenged mice in (C) (tumor‐bearing mice for control, tumor‐free mice from combination therapy (*n* = 3 for each group)) were co‐cultured with KPC tumor cells. After 24 h of co‐culture, whole cells were separated from the medium and cultured for another 24 h on ELISPOT plates pretreated with anti‐IFN‐γ antibodies. After removing all cells, secreted IFN‐γs were visualized as spots. The bar represents 1 mm. Data are presented as mean ± SEM. Student's *t*‐test was performed. *P < 0.05, **P < 0.01, ***P < 0.001, ****P < 0.0001.

To evaluate the effects of combination therapy in the human PDAC mimetic model, the tumor growth rate in both unilateral and bilateral KPC tumor models was examined. After administering C‐REV and 2′3′‐cGAMP therapy twice on a unilateral KPC tumor model in a span of 2 weeks, the tumor growth was significantly inhibited (Fig. 5A). Out of the seven mice treated with combination therapy, four showed a complete response (CR), and only two out of eight mice showed CR after C‐REV monotherapy. Even with only a single set of C‐REV and 2′3′‐cGAMP treatment in a bilateral tumor model, combination therapy significantly suppressed tumor growth in both flanks (Fig. 5B). To assess the long‐term persistence of antitumor immunity, mice that exhibited CR after C‐REV treatment or combination therapy in Fig. 5A were rechallenged with cultured KPC cells 3 months after the eradication of tumors. All previously cured mice rejected tumors within 4 weeks, whereas nine of 10 age‐matched tumor‐naive control mice exhibited tumor growth (Fig. 5C).

TIL analyses on a unilateral KPC tumor model 6 days after treatment administration also revealed that combination therapy significantly increased unexhausted PD1‐low TIM3‐low CD8^+^ T‐cells (Fig. 5D–F). In addition, only combination therapy‐treated tumors demonstrated an increase in the KLRG1^+^ population among PD1‐low CD8^+^ T‐cells (Fig. 5G,H, Fig. S3A,B,D). Aside from improving the quality and number of tumor‐infiltrated CD8^+^ T‐cells, combination therapy also increased the population of inflammatory DC (CD11c^+^Ly6c^+^) (Fig. 5I), CD103^+^ DC (Fig. 5J,K) and XCR1^+^ DC (Fig. S3C). TDLNs from combination‐treated mice and 2′3′‐cGAMP‐treated mice also contained more CD103^+^ XCR^+^ CD8^+^ DC (Fig. 5K), implying that 2′3′‐cGAMP treatment facilitated the activation of DC and promoted the migration of DC toward TDLN. Alongside this, only combination therapy was able to increase significantly CD44^+^ CD8^+^ T‐cells in the TDLN (Fig. 5L), suggesting that combination therapy enabled the efficient priming of naïve CD8^+^ T‐cells in TDLN. Overall, these results demonstrate that combination therapy has the potential to activate DC and promote the migration of DC toward the TDLN to present tumor tissue‐derived antigens.

Since GM‐CSF‐armed oncolytic viruses have been reported to induce DC differentiation, we hypothesized that GM‐CSF‐armed OV combined with 2′3′‐cGAMP could elicit even greater antitumor effects. To test this, we utilized a newly generated C‐REV armed with murine GM‐CSF in the UL43 region. The combination of C‐REV‐GM‐CSF with 2′3′‐cGAMP exerted almost the same effect on tumor growth and TIL as the combination of C‐REV with 2′3′‐cGAMP did (Fig. 5B,G,H, Fig. S3B,C). However, after 48 h of co‐culturing lymphocytes from TDLN and KPC tumor cells *in vitro* with different effector‐to‐target (ET) ratios, the highest cytotoxic effect at the ET 1 ratio was generated by lymphocytes from the C‐REV‐GM‐CSF with 2′3′‐cGAMP combination therapy on day 6 (Fig. 5M). The same tendency was observed even when lymphocytes from TDLN on day 22 were used, although the differences among the groups were less pronounced (Fig. S3E). This result suggests that C‐REV‐GM‐CSF combination therapy could be marginally better than C‐REV in inducing tumor‐antigen‐specific CD8^+^ T‐cells.

Additionally, it prompted us to confirm further the ability of C‐REV with 2′3′‐cGAMP to induce long‐term tumor antigen‐specific immune memory. To reactivate the antitumor immunity, we subcutaneously injected 1 × 10^5^ of IFN‐β pre‐exposed KPC cells to the CR mice in Fig. 5C 2 days before dissecting the TDLN. An LDH assay using the supernatant from the co‐culture of the TDLN lymphocytes with KPC cells revealed that in the ET 1 ratio, lymphocytes from the CR mice of the combination therapy group showed higher cytotoxicity than did the tumor‐bearing control mice from the same rechallenge experiment (Fig. S3F). Similarly, results from the IFN‐γ ELISPOT assay of the co‐cultured lymphocytes displayed higher numbers of spots in CR mice from combination therapy than in control mice (Fig. 5N). Taken together, these results strongly suggest that combination therapy restimulates the dormant antitumor immunity by enhancing the tumor antigen presentation toward tumor‐draining lymph nodes and facilitating immune memory formation against tumor antigens.

## 4 Discussion

Among the currently developing immune therapies, OV therapy is regarded as unconventional mainly because it not only eliminates tumor cells through viral lysis but also evokes viral immune responses capable of transforming a ‘cold’ immuno‐suppressive TME to a ‘hot’ inflamed and immunogenic TME [35]. However, the immune responses toward OV facilitate the clearance of both tumor and viral infection. As such, to achieve persistent and stronger antitumor efficacy, it is crucial that the activation of antiviral immunity rapidly and substantially reinforces antitumor immunity. In this study, by leveraging the differential expression of 2′3′‐cGAMP receptors among lymphocytes and tumor cells, we proposed a combination therapy of C‐REV with 2′3′‐cGAMP for enhanced antitumor immunity. Our results clearly demonstrated that the tumor cells used in this study were unable to uptake 2′3′‐cGAMP and, consequently, unable to activate the antiviral STING pathway (Fig. 2A). However, the tumor cells were still responsive to IFN. Considering that endothelial cells can release IFN upon uptake of 2′3′‐cGAMP, it is possible for nearby tumor cells to suppress OV replication [36]. To circumvent this possibility, we treated tumors with 2′3′‐cGAMP 2 days after C‐REV treatment. In doing so, C‐REV had already proceeded to the advanced stages of viral replication by the time 2′3′‐cGAMP was introduced. Whether or not introducing 2′3′‐cGAMP at a later time point can give better antitumor effects remains to be explored.

Numerous synthetic STING agonists have emerged over the years due to the limitations of using the natural STING agonist, 2′3′‐cGAMP. One major limitation of 2′3′‐cGAMP is its rapid degradation by phosphodiesterases abundant in the extracellular matrix, particularly ENPP1 (ecto‐nucleotide pyrophosphatase phosphodiesterase) [37]. Consequently, we decided to explore combining C‐REV with ADU‐S100, a STING agonist resistant to phosphodiesterases [38]. Although ADU‐S100 had strong tumor‐suppressive effects when combined with C‐REV, we found that very few TIL survived in the tumor site, resulting in a much less abscopal effect than 2′3′‐cGAMP. Since OV alone is already regarded as a strong stimulus for lymphocytes and since STING agonists have also been reported to induce overactivation in lymphocytes and, eventually, apoptosis, we suspect that the lack of TIL after ADU‐S100 combination therapy can be attributed to lymphocyte overactivation [39]. Utilizing these observations, we speculate that ADU‐S100 combination therapy can be as good as, if not better than, 2′3′‐cGAMP combination therapy once the dose of ADU‐S100 is optimized. When optimizing its dose, the lymphocyte‐stimulating effects of the OV present should be considered. Hence, doses lower than what are typically used for a single treatment would be recommended.

It has been widely reported that PDAC possesses a strong immunosuppressive TME that not only limits the numbers of infiltrating CD8^+^ T‐cells but also rapidly induces markers of exhaustion, including CD69, PD1, TIM3, TIGIT on the infiltrating CD8^+^ T‐cells [40]. We have previously reported that C‐REV induced the infiltration of PD1‐low CD8^+^ T‐cells [19]. Consistent with our previous results, our TIL analysis revealed that C‐REV treatment increased the infiltration of PD1‐low and CD69‐low CD8^+^ T‐cells on SCCVII tumors and PD1‐low and TIM3‐low CD8^+^ T‐cells in both Pan02 and KPC tumors. Although a limited expression of PD1 and TIM3 suggests that C‐REV mainly recruits unexhausted CD8^+^ T‐cells, a limited expression of CD69 also indicates that these CD8^+^ T‐cells may have not yet been fully activated or differentiated to effector CD8^+^ T‐cells. 2′3′‐cGAMP seemed to have effectively circumvented this limitation by promoting CD8^+^ T‐cell terminal differentiation via the activation of tumor‐infiltrating DC, given that the combination therapy recruited CD8^+^ T‐cells whose expression is not only low in PD1 but also high in KLGR1, a terminal differentiation marker for effector CD8^+^ T‐cells [40]. Existing literature suggests that PD1‐low KLRG1^+^ CD8^+^ T‐cells can function as an activated effector CD8^+^ T‐cells while also being resistant to the immunosuppressive effects exerted by the PDL1‐enriched TME [41].

Activation markers found on the DC we analyzed suggest that combination treatment increased the number of functional DC, particularly antigen‐presenting DC, in both the tumor tissue and TDLN. Within the tumor tissue, DC activated by 2′3′‐cGAMP and carrying the tumor antigen can either migrate to the TDLN to prime tumor‐antigen‐specific CD8^+^ T‐cells or remain in the tumor site. We speculate that the activated DC that remained in the tumor site account for the observed intra‐tumoral KLRG1^+^ CD8^+^ T‐cells. These resident DC could have promoted the proliferation and terminal differentiation of the primed CD8^+^ T‐cells, which were newly recruited to the tumor site during OV‐induced inflammation, by presenting tumor antigens and costimulatory molecules and secreting the necessary inflammatory cytokines. Through the concerted effort of C‐REV, which recruits newly‐infiltrated – unexhausted PD1‐low – CD8^+^ T‐cells, and 2′3′‐cGAMP, which constantly activates CD8^+^ T‐cells against tumor antigens, the immunosuppressive ‘cold’ TME could be transformed to a ‘hot’ immunogenic TME. Persistent antitumor, rather than antiviral, immune memory formation after combination therapy was clearly demonstrated in the abscopal effects observed in the non‐injected flank, along with the results of the rechallenge experiments and ELISPOT assay. The observation that TDLN from combination‐treated mice had higher cytotoxic capability toward tumor cells (Fig. 5N) strongly suggests that combination therapy enhanced the DC population that specifically cross‐presents tumor, rather than viral, antigens.

Although GM‐CSF and 2′3′‐cGAMP have overlapping roles in DC activation, 2′3′‐cGAMP alone might have allowed DC to reach their maximum activation status, thus rendering GM‐CSF ineffective and preventing the combination of GM‐CSF armed C‐REV with 2′3′‐cGAMP to provide additional benefit compared with that of unarmed C‐REV. Rather than enhancing the effect of 2′3′‐cGAMP on DC activation in the TME, GM‐CSF could have played only a redundant role.

In this study, we limited our scope to determining mechanisms of immune responses evoked only by combining C‐REV with 2′3′‐cGAMP and, to some extent, ADU‐S100. Combination therapy using other STING agonists and other types of OVs was not explored in this study. In addition, an in‐depth analysis of how the kinetics of intracellular processes related to the viral replication machinery were affected by 2′3′‐cGAMP treatment is well beyond the aim of this study.

## 5 Conclusion

By demonstrating that the immunosuppressive PDAC TME can be transformed into an immunogenic TME with increased numbers of tumor‐antigen specific and unexhausted CD8^+^ T‐cells, combination therapy shows its potential in increasing the sensitivity of PDAC to different types of immune checkpoint inhibitors (ICIs). Overall, this study paves the way for exploring the clinical applications of STING agonists in combination with OVs and ICIs in eradicating PDAC.

## Conflict of interest

Maki Tanaka is a Takara Bio, Inc. employee, and Hideki Kasuya received research funding from Takara Bio, Inc. The other authors declare no conflict of interest. Takara Bio, Inc. had no role in the design of the study, in the collection, analysis or interpretation of data, in the writing of the article or in the decision to publish the article.

## Author contributions

Conceptualization: SM, YN, HK. Investigation: SM, PAS, IRE, TI. Funding acquisition: SM, YN, HK. Project administration: HK. Supervision: MT, HK. Writing – original draft: SM, PAS. Writing – review & editing: PAS, IB‐V, DM, MA, MAMA, NM. Article approval: all authors.

### Peer review

The peer review history for this article is available at https://www.webofscience.com/api/gateway/wos/peer‐review/10.1002/1878‐0261.13603.

## Supporting information


**Fig. S1.** ICP34.5 partially inhibited TBK1 phosphorylation.

**Fig. S2.** C‐REV with ADU‐S100 had limited abscopal effect.

**Fig. S3** C‐REV with cGAMP enhanced antitumor immune responses.

**Table S1.** The three STR profiles were analyzed through ATCC's STR authentication service of murine cell lines.

## Data Availability

Relevant information regarding data collected and materials used will be provided upon reasonable request.
