# Supplementary material for: STING activator 2′3′‐cGAMP enhanced HSV‐1‐based oncolytic viral therapy
Source: Mol Oncol. 2024 Feb 23;18(5):1259–77. doi: 10.1002/1878-0261.13603 (PMC11076993; doi:10.1002/1878-0261.13603)
Supplement: Supplementary file 1 — Fig. S1. ICP34.5 partially inhibited TBK1 phosphorylation. [file MOL2-18-1259-s002.pdf]

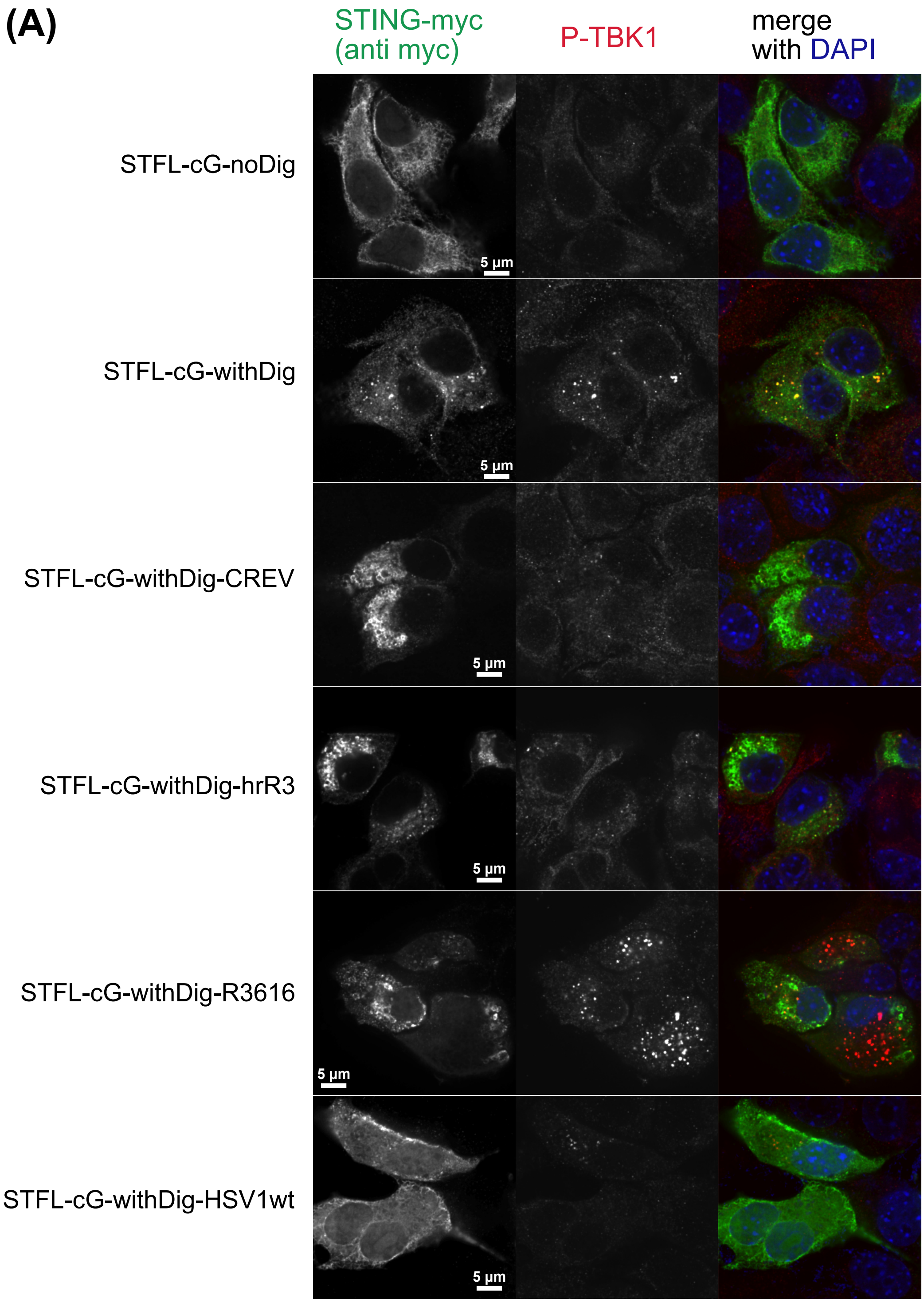

FIGURE S1

ICP34.5 partially inhibited TBK1 phosphorylation. (A) The representative images of immunofluorescent cell staining. KPC cells which stably express human STING-full length (STFL) were cultured in coverslips and treated with 2'3'-cGAMP (5µM) in the presesnce or in the absence of Digitionin as indicated. After 30 minutes, cells were infected with the corresponding viruses at MOI 5. At 3 hpi, cells were fixed with formaldehyde, permeabilized with MeOH, and treated with the indicated primary antibodies.
