# Supplementary material for: STING activator 2′3′‐cGAMP enhanced HSV‐1‐based oncolytic viral therapy
Source: Mol Oncol. 2024 Feb 23;18(5):1259–77. doi: 10.1002/1878-0261.13603 (PMC11076993; doi:10.1002/1878-0261.13603)
Supplement: Supplementary file 2 — Fig. S2. C‐REV with ADU‐S100 had limited abscopal effect. [file MOL2-18-1259-s001.pdf]

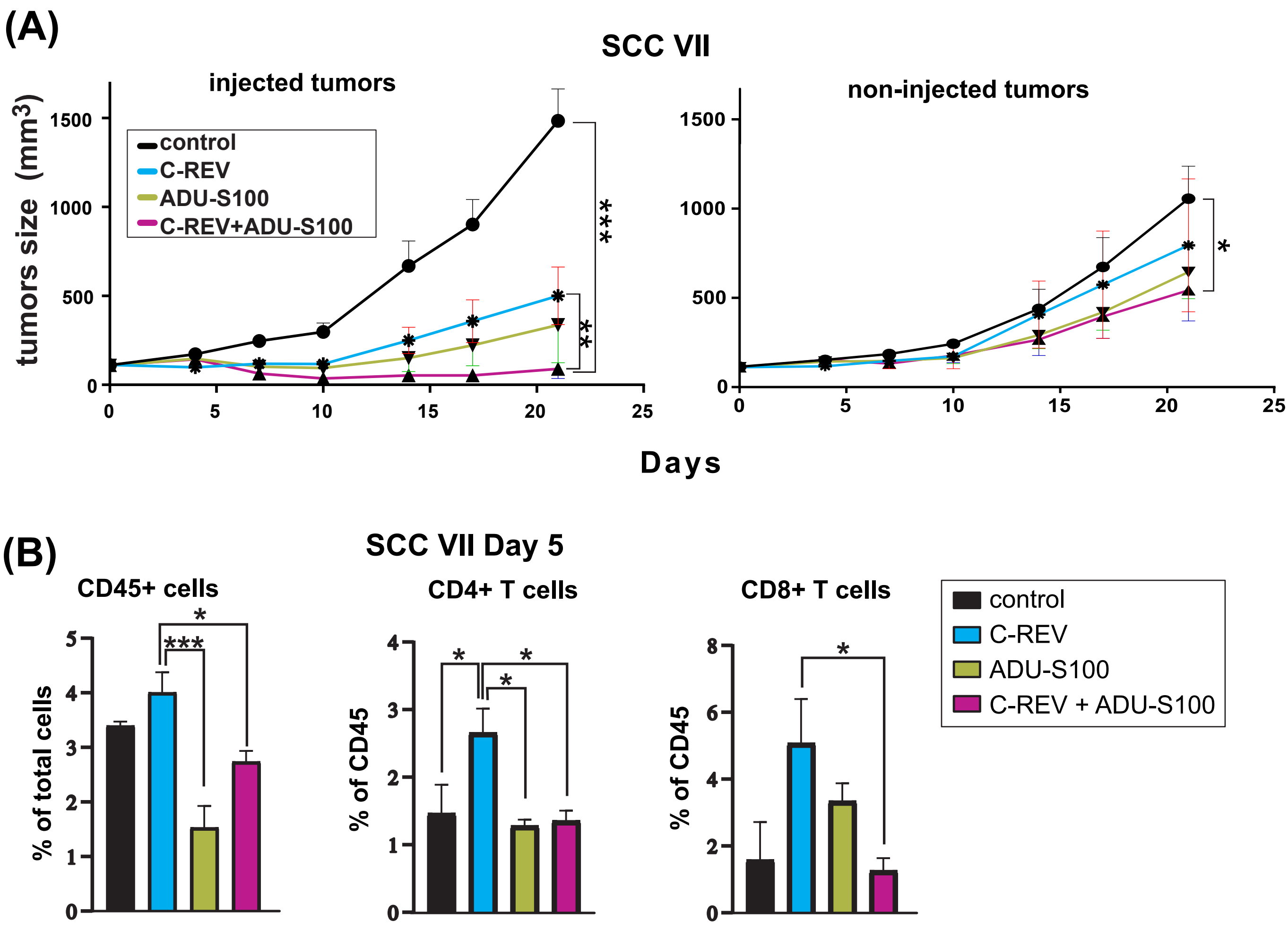


**FIGURE S2**

C-REV with ADU-S100 had limited abscopal effect. (A) Tumor growth curves in SCCVII bilateral tumor model. C3H mice were inoculated with tumors. On Day 0, tumors on one side was injected with C-REV intratumorally (IT) and the same tumor was injected with ADU-S100 (8 μg) intratumorally on Days 3 and 6. Data are presented as mean ± SEM (n = 4 mice). Two-way ANOVA with Dunnett's post-test was performed. (B) Flow analysis on the tumor infiltrated lymphocytes. SCCVII tumors were treated with C-REV on Day 0 and/or ADU-S100 on Day 3 as indicated and harvested on Day 5. The tumor tissues were then digested enzymatically, and stained for surface markers. CD4+ T cells were selected as CD45+CD3+CD4+ cells while CD8+ T cells were selected as CD45+CD3+CD8+ cells. Data are presented as mean ± SEM (n = 3 mice). One-way ANOVA with Dunnett's multiple comparison tests were performed.

* $p < 0.05$, ** $p < 0.01$, *** $p < 0.001$.
