# Supplementary material for: STING activator 2′3′‐cGAMP enhanced HSV‐1‐based oncolytic viral therapy
Source: Mol Oncol. 2024 Feb 23;18(5):1259–77. doi: 10.1002/1878-0261.13603 (PMC11076993; doi:10.1002/1878-0261.13603)
Supplement: Supplementary file 3 — Fig. S3 C‐REV with cGAMP enhanced antitumor immune responses. [file MOL2-18-1259-s004.pdf]

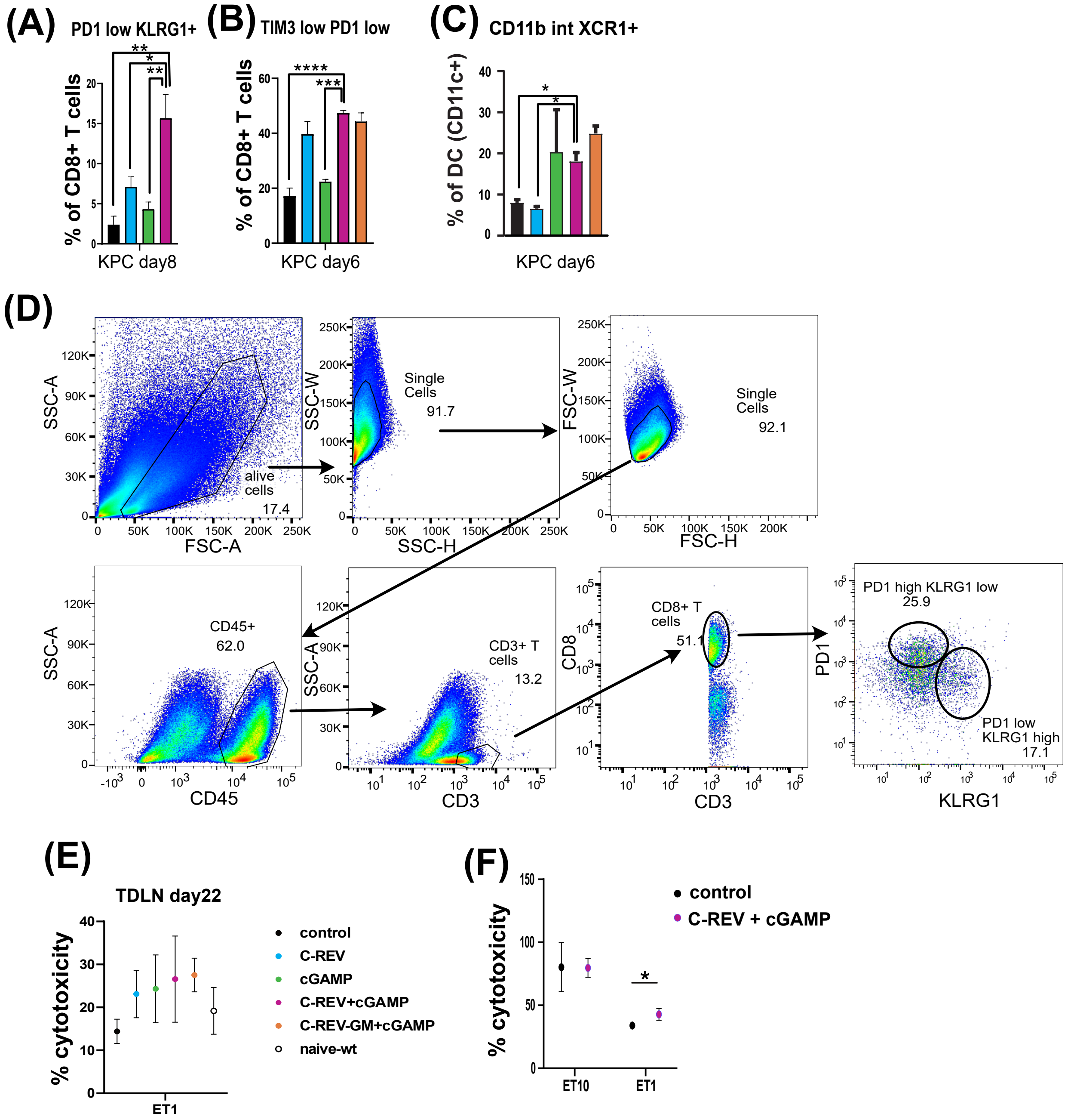

FIGURE S3

C-REV with cGAMP enhanced anti-tumor immune responses. (A) Flow analysis on tumor infiltrated lymphocytes (TILs). KPC tumors were harvested on Day8, digested enzymatically, and stained for surface markers. CD8+ T cells were then selected as CD45+CD3+CD8+ cells. (B and C) Flow analysis on TILs. KPC tumors were harvested on Day6, digested enzymatically, and stained for surface markers. CD8+ T cells were selected as CD45+CD3+CD8+ cells and then analyzed by their surface expression of TIM3 and PD1. DCs were selected as CD45+CD11b+MHC-classII highCD11c+ cells and then analyzed through their surface expression of XCR1. Data are presented as mean ± SEM (n = 3 mice). Dunnett's multiple comparison tests were performed. (D) Gating strategy. We followed a commonly employed gating method for selecting CD3+CD8+ T cells from a digested tumor sample. (E and F) Cytotoxicity assay involving lymphocytes from TDLN and KPC cells. (E) The TDLN were harvested on Day22 from KPC tumor-bearing mice at the end of the tumor growth curve experiment. Whole lymphocytes from each TDLN were co-cultured with KPC tumor cells with the indicated ET ratio in IL2-containing medium for 24 hrs. The medium from the co-culture was collected and LDH levels were measured to assess the anti-tumor cytotoxicity of the lymphocytes. Data are presented as mean ± SEM (n = 3 mice). Each ET ratio was performed with triplicates. Naive-wt mice which has never been inoculated with tumor was used as a negative control. (F) Combination therapy-treated mice from the KPC rechallenge experiment were injected with $1x10^5$ IFNa-treated KPC cells subcutaneously and 3 days after, the TDLNs were harvested. Whole lymphocytes from the lymph nodes were co-cultured with KPC tumor cells. The co-cultured medium was then used to evaluate the cytotoxicity of the lymphocytes as in (E). Data are presented as mean ± SEM. Student's t-test was performed. * $p < 0.05$, ** $p < 0.01$, *** $p < 0.001$, **** $p < 0.0001$.
