# Supplementary material for: STING activator 2′3′‐cGAMP enhanced HSV‐1‐based oncolytic viral therapy
Source: Mol Oncol. 2024 Feb 23;18(5):1259–77. doi: 10.1002/1878-0261.13603 (PMC11076993; doi:10.1002/1878-0261.13603)
Supplement: Supplementary file 4 — Table S1. The three STR profiles were analyzed through ATCC's STR authentication service of murine cell lines. [file MOL2-18-1259-s003.docx]

| **STR PROFILE REPORTS** | | | | | |
| --- | --- | --- | --- | --- | --- |
| **Locus** | **Pan-02** | **SCC-VII** | | **KPC** | |
| **18-3** | 15 | 16 |  | 16 | 18 |
| **4-2** | 20.3 | 18.3 | 19.3 | 18.3 | 20.3 |
| **6-7** | 17 | 12 |  | 18 |  |
| **19-2** | 13 | 12 |  | 13 |  |
| **1-2** | 19 | 16 |  | 19 |  |
| **7-1** | 26.2 | 26 |  | 27.2 |  |
| **1-1** | 17 | 10 |  | 16 | 17 |
| **3-2** | 14 | 13 | 14 | 14 |  |
| **8-1** | 16 | 16 | 17 | 16 |  |
| **2-1** | 16 | 9 |  | 9 | 16 |
| **15-3** | 22.3 | 25.3 |  | 21.3 | 22.3 |
| **6-4** | 18 | 18 |  | 18 |  |
| **11-2** | 16 | 16 | 17 | 16 |  |
| **17-2** | 16 | 14 | 15 | 16 |  |
| **12-1** | 17 | 15 | 16 | 17 |  |
| **5-5** | 16 | 14 | 15 | 17 |  |
| **X-1** | 29 | 26 | 27 | 28 |  |
| **13-1** | 16 | 17 | 18 | 15.2 | 18 |

Supplementary Table 1. The three STR profiles were analyzed through ATCC’s STR authentication service of murine cell lines.
